# Prevalence and Outcomes of Unilateral Versus Bilateral Oophorectomy in Women With Ovarian Cancer: A Population-Based Study

**DOI:** 10.3389/fonc.2022.866443

**Published:** 2022-07-08

**Authors:** Jiaqiang Xiong, Zhuoqun Zhang, Yanyan Liu, Guanlan Fan, Kejia Wu, Wei Zhang

**Affiliations:** ^1^ Department of Obstetrics and Gynecology, Zhongnan Hospital of Wuhan University, Wuhan, China; ^2^ Department of Obstetrics and Gynecology, The Central Hospital of Wuhan, Tongji Medical College, Huazhong University of Science and Technology, Wuhan, China

**Keywords:** ovarian cancer, prevalence, outcomes, population-based study, SEER, unilateral oophorectomy, bilateral oophorectomy

## Abstract

**Background:**

Unilateral oophorectomy has the benefits of preserving the ovarian function of fertility and hormone secretion, but the precise inclusion criteria for candidates for this procedure remain controversial. This study aimed to compare the prevalence and therapeutic efficiency of unilateral oophorectomy in women with ovarian cancer who underwent bilateral oophorectomy; moreover, it aimed to identify the appropriate candidates for unilateral oophorectomy.

**Methods:**

Female patients diagnosed with stage I-III ovarian cancer between 2000 and 2017 were retrospectively identified from the Surveillance, Epidemiology, and End Results program database. Overall survival (OS) and disease-specific survival (DSS) after unilateral or bilateral (salpingo-) oophorectomy were estimated. Cumulative mortality rates (CMRs) for non-cancer comorbidities were also estimated.

**Results:**

A total of 28,480 women with ovarian cancer were included in this study, of whom 11,517 died during the study period. Of the patients, 7.5% and 48.0% underwent unilateral and bilateral oophorectomy, respectively. Overall, for stage-Ia tumors, unilateral oophorectomy was associated with remarkably better OS and DSS than bilateral oophorectomy (OS: p < 0.001; DSS: p = 0.01). For stage-Ib and stage-Ic ovarian tumor, there was no significant difference between the OS and DSS of patients treated by unilateral oophorectomy and those treated by bilateral oophorectomy. For stage-II and stage-III ovarian cancer, unilateral oophorectomy was associated with remarkably worse OS and DSS than bilateral oophorectomy. Among the reproductive-age women younger than 50 years, the OS and DSS of patients with stage-I tumors receiving unilateral oophorectomy were comparable to those receiving bilateral oophorectomy, even for high-grade stage-Ic tumors (all p > 0.05). For those aged 50 years and older, OS and DSS of patients with stage-I tumor receiving unilateral oophorectomy were significantly worse than those receiving bilateral oophorectomy, even for low-grade stage-Ia ovarian tumor (OS: p < 0.001; DSS: p = 0.02).

**Conclusion:**

Unilateral oophorectomy exhibited excellent oncological superiority and was equivalent to bilateral oophorectomy for stage-I ovarian tumors among women of reproductive age. For women of reproductive age, the criteria of unilateral oophorectomy can be appropriately broadened to high-grade stage-Ic diseases because of the better performance of unilateral oophorectomy in this population.

## Introduction

Ovarian cancer is the sixth leading cause of global cancer deaths, accounting for over 294,000 new cancer cases and 198,000 new cancer deaths worldwide in 2019 (1). Ovarian cancer ranks fifth in cancer deaths among American women, accounting for more deaths than any other female reproductive system cancer. A woman’s risk of developing ovarian cancer during her lifetime is about 1 in 78, and her lifetime chance of dying from ovarian cancer is approximately 1 in 108 (2). Although the 5-year relative-survival rate of localized ovarian cancer can reach 93%, only 16% of the cases have the opportunity to be diagnosed at an early stage (3). Of the tumors, 57% were accompanied by distant metastases at the time of cancer diagnosis, implying a particularly unfavorable prognosis with a survival rate of only 30% (3, 4). As a consequence of the low early detection, as well as the relatively high malignant potential, the overall 5-year relative survival rate generally ranges between 30% and 40% across the globe (5). Strikingly, only very modest increases have been achieved (2%–4%) since 1995 (5). Therefore, despite its significance to public health, the etiology of this lethal disease is not completely understood.

The standard treatment for ovarian cancer includes upfront surgery to accurately diagnose and stage the disease and perform maximal cytoreduction, followed by taxanes and platinum–based combination chemotherapy in most patients (6). Traditionally, surgical staging of ovarian cancer has included exploratory laparotomy with peritoneal washings, hysterectomy, salpingo-oophorectomy, omentectomy, multiple peritoneal biopsies, and potential pelvic and para-aortic lymphadenectomy.

When preservation of fertility is desired and the disease seems confined to a single ovary, preservation of the uterus and contralateral ovary is often possible (6). Compared with bilateral oophorectomy, unilateral oophorectomy can preserve the other side of the ovary to maintain the ovarian function of fertility and hormone secretion. The major concerns of unilateral oophorectomy focused on the potential risks of residual tumor, tumor recurrence, and a newly occurring tumor on the other side of the ovary (7, 8). Therefore, it is important to determine which proportion of patients are suitable and/or have the opportunity to receive unilateral oophorectomy and preserve the other side of the ovary.

This study aimed to characterize the prevalence and outcomes of unilateral oophorectomy in women with ovarian cancer and compare it with bilateral oophorectomy to distinguish the appropriate proportion of patients with ovarian cancer to be treated with unilateral oophorectomy. The results will guide researchers and clinicians in determining the optimal therapy for patients with ovarian cancer.

## Materials and Methods

### Data Sources and Study Population

This retrospective cohort study used data from the Surveillance, Epidemiology, and End Results (SEER) program. The SEER database is a population-based cancer registry covering nearly 30% of the US population and collecting cancer demographics, incidence, survival, and treatment data. The SEER*Stat software version 8.3.8 was used for the analysis (9). This study was performed according to the STROCSS guidelines (Strengthening the reporting of cohort, cross-sectional and case-control studies in surgery) (10).

Female patients diagnosed with the first primary malignant ovarian cancer (site codes: C56.9) between 2000 and 2017 were extracted from the SEER 18 database (2020 submission) (11). Only patients with unilateral-originated ovarian cancer were included because patients with bilateral origin might have lost their opportunity to undergo unilateral oophorectomy. Patients diagnosed only through autopsy or death certificates were excluded. We further excluded patients without complete follow-up information, including follow-up duration and age at diagnosis. To accurately evaluate the effects of surgical operations, we further excluded patients with advanced-stage cancer or those with a cancer of unknown stage (**Figure S1**).

Since it is a publicly available database, access to the SEER data required a signed research data agreement form. The Institutional Review Board of Zhongnan Hospital of Wuhan University waived the institutional review board approval for the data obtained from the SEER database, as the study did not directly involve human subjects, and all data were anonymized. The requirement for informed consent was waived.

### Definition of Variables

All patients were followed between the time of the first primary diagnosis of ovarian cancer and the time of their death, exiting the study alive, or the end of the study (December 31, 2017). Among the patients included in this study, we evaluated the following variables: age at diagnosis, race, year of diagnosis, cancer stage, American Joint Committee on Cancer Staging (AJCC) N stage, AJCC T stage, surgical therapy, cause of death, histological types, urban/rural residency at diagnosis, median household income, follow-up time, and vital status at the end of follow-up.

As the SEER database records the survival duration in months, and a month was the shortest time interval available for analysis, survival durations shorter than 1 month were recorded as 0 months in the SEER program. Age at cancer diagnosis was divided into 4 groups for comparison: “15-39 years,” “40-59 years,” “60-79 years,” and “80+ years”. Patients aged 15-49 years were selected for specific analyses, as this proportion of women had a greater desire for fertility preservation.

For ovarian cancer, the SEER program derived TNM values of the stage from the International Federation of Gynecology and Obstetrics (FIGO) stage. Thus, FIGO information of this study was inferred from TNM-stage values. TNM-stage values were extracted from AJCC 3^rd^ stage codes for patients diagnosed between 2000 and 2003, AJCC 6^th^ stage codes for patients diagnosed between 2004 and 2009, AJCC 7^th^ stage codes for patients diagnosed between 2010 and 2015, and SEER combined stage for patients diagnosed in 2016 and 2017 (12). We excluded the patients with stage IV ovarian cancer.

The SEER program provided detailed site-specific surgical information for the included patients (13–15). Surgical operations for ovarian cancer were divided into two major groups: unilateral and bilateral oophorectomy. To avoid confusion, local excision/destruction and unknown surgical operations were excluded (surgery codes: 17 and 90-99). Unilateral oophorectomy was defined as total removal of the tumor or (single) ovary, and unilateral (salpingo-) oophorectomy with or without hysterectomy (surgery codes: 25-28 and 35-37). Bilateral oophorectomy includes bilateral (salpingo-) oophorectomy with or without hysterectomy, cytoreductive surgery, and pelvic exenteration (surgery codes: 60-74). Surgical operations with unknown laterality were excluded for accuracy (surgery codes: 55-57 and 80) (13).

Causes of death of patients with ovarian cancer were classified into two major groups: death from cancer (i.e., a second primary cancer) and death from non-cancer comorbidities (i.e., deaths from any medical cause other than cancer). Causes of death were defined by the SEER cause-specific death classification variable from death certificates (15–17). Non-cancer causes were categorized into 26 major groups. These groups were further divided into seven broad categories: infectious diseases, cardiovascular diseases (CVD), respiratory diseases, gastrointestinal and liver diseases, renal diseases, external injuries, and other non-cancer causes.

### Statistical Analysis

We estimated the characteristics of the patients with ovarian cancer. Trends in surgical operations were characterized by age at diagnosis and year of diagnosis. Moreover, we analyzed the overall survival (OS) and disease-specific survival (DSS) of patients using the Kaplan-Meier method. The OS rate was defined as the percentage of survivors (all causes of death) after follow-up. The DSS rate was defined as the percentage of patients who have not died from ovarian cancer (rather than from other causes) in a defined period of time (18). Cox regression models were used to assess the significance of differences in the OS and DSS analyses. The cumulative mortality rate (CMR) was estimated for non-cancer comorbidities (15).

All analyses were performed using SEER*Stat software version 8.3.8 (9) and R 3.6.3 (19). Tests were two-tailed, with a p-value of less than 0.05 considered statistically significant.

## Results

### Baseline Characteristics

In this population-based study involving 28,480 women with stage I-III ovarian cancer, 11,517 (40.4%) deaths were recorded, with a median follow-up time of 4.1 years (range: 0–17.9 years) (**Figure S1** and **Table 1**). Most of the patients were aged 40–79 years (83.3%) and were white (82.6%). Of the cancers, 46.8% were stage-I tumors. Serous ovarian cancer accounted for the majority of tumors (51.9%), followed by endometrioid carcinoma (20.7%) (**Table 1**).

**Table 1:** Characteristics of patients included in this study.

Characteristics	No. of patients (%)	No. of deaths (%)	Surgical procedure	
			Unilateral oophorectomy (%)	Bilateral oophorectomy (%)
Total	28,480 (100%)	11,517 (100%)	2,145 (100%)	13,678 (100%)
Age				
15-39	2,497 (8.8%)	366 (3.2%)	842 (39.3%)	568 (4.2%)
40-59	12,174 (42.7%)	3,634 (31.6%)	678 (31.6%)	5,743 (42%)
60-79	11,549 (40.6%)	5,761 (50%)	466 (21.7%)	6,269 (45.8%)
80+	2,260 (7.9%)	1,756 (15.2%)	159 (7.4%)	1,098 (8%)
Race				
White	23,532 (82.6%)	9,716 (84.4%)	1,674 (78%)	11,417 (83.5%)
AI/AN	179 (0.6%)	74 (0.6%)	14 (0.7%)	89 (0.7%)
API	2,838 (10%)	818 (7.1%)	247 (11.5%)	1,272 (9.3%)
Black	1,806 (6.3%)	898 (7.8%)	190 (8.9%)	858 (6.3%)
Unknown	125 (0.4%)	11 (0.1%)	20 (0.9%)	42 (0.3%)
Hispanic origin				
Non-Hispanic	25,045 (87.9%)	10,385 (90.2%)	1,759 (82%)	12,101 (88.5%)
Hispanic	3,435 (12.1%)	1,132 (9.8%)	386 (18%)	1,577 (11.5%)
Year of diagnosis				
2000-2009	15,283 (53.7%)	8,025 (69.7%)	1,292 (60.2%)	7,021 (51.3%)
2010-2017	13,197 (46.3%)	3,492 (30.3%)	853 (39.8%)	6,657 (48.7%)
Rural/urban status				
Urban	3,064 (10.8%)	1,431 (12.4%)	204 (9.5%)	1,497 (10.9%)
Rural	25,392 (89.2%)	10,071 (87.4%)	1,939 (90.4%)	12,169 (89%)
Unknown	24 (0.1%)	15 (0.1%)	2 (0.1%)	12 (0.1%)
Median house-hold income^1^				
Low	370 (1.3%)	166 (1.4%)	33 (1.5%)	171 (1.3%)
Median	18,925 (66.5%)	7,859 (68.2%)	1,500 (69.9%)	9,134 (66.8%)
High	9,184 (32.2%)	3,491 (30.3%)	612 (28.5%)	4,372 (32%)
Unknown	1 (0.004%)	1 (0.009%)		1 (0%)
FIGO stage				
Stage Ia	8,314 (29.2%)	1,528 (13.3%)	1,138 (53.1%)	2,990 (21.9%)
Stage Ib	145 (0.5%)	38 (0.3%)	9 (0.4%)	57 (0.4%)
Stage Ic	4,551 (16%)	989 (8.6%)	416 (19.4%)	1,636 (12%)
Stage I, NOS	331 (1.2%)	90 (0.8%)	55 (2.6%)	103 (0.8%)
Stage IIa	1,165 (4.1%)	402 (3.5%)	68 (3.2%)	518 (3.8%)
Stage IIb	1,607 (5.6%)	599 (5.2%)	79 (3.7%)	813 (5.9%)
Stage IIc	1,308 (4.6%)	550 (4.8%)	62 (2.9%)	602 (4.4%)
Stage II, NOS	210 (0.7%)	120 (1%)	17 (0.8%)	102 (0.7%)
Stage IIIa	780 (2.7%)	386 (3.4%)	28 (1.3%)	389 (2.8%)
Stage IIIb	1,127 (4%)	617 (5.4%)	35 (1.6%)	681 (5%)
Stage IIIc	6,613 (23.2%)	4,400 (38.2%)	153 (7.1%)	4,544 (33.2%)
Stage III, NOS	2,329 (8.2%)	1,798 (15.6%)	85 (4%)	1,243 (9.1%)
Histology				
Clear cell	3,771 (13.2%)	1,138 (9.9%)	191 (8.9%)	1,575 (11.5%)
Endometrioid	5,908 (20.7%)	1,446 (12.6%)	439 (20.5%)	2,581 (18.9%)
Mucinous	4,022 (14.1%)	1,074 (9.3%)	680 (31.7%)	1,379 (10.1%)
Serous	14,779 (51.9%)	7,859 (68.2%)	835 (38.9%)	8,143 (59.5%)
Grade				
Grade I	3,808 (13.4%)	675 (5.9%)	492 (22.9%)	1,437 (10.5%)
Grade II	5,202 (18.3%)	1,740 (15.1%)	410 (19.1%)	2,272 (16.6%)
Grade III	8,511 (29.9%)	4,562 (39.6%)	372 (17.3%)	4,742 (34.7%)
Grade IV	4,448 (15.6%)	1,905 (16.5%)	155 (7.2%)	2,671 (19.5%)

AI/AN, American Indian/Alaska Native; API, Asian or Pacific Islander.

^1^Low income referred to those with a median house-hold income of less than $35,000. Median income referred to those with a median house-hold income ranged from $35,000 to $75,000. High income referred to those with a median house-hold income of more than $75,000.

**Figure 1:**
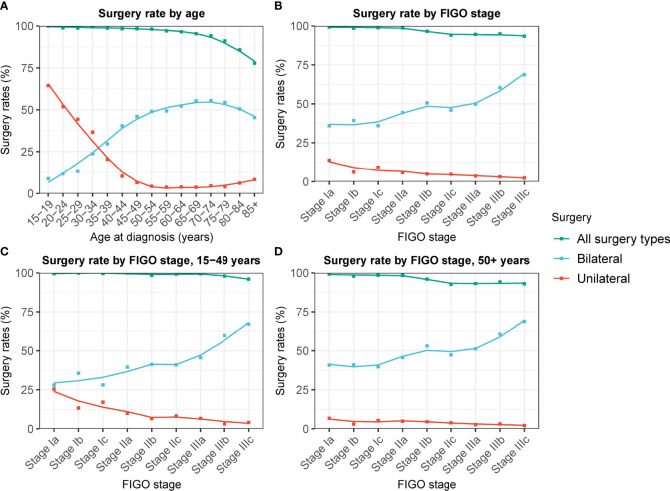
Changes in unilateral and bilateral oophorectomy rate of patients with ovarian cancer by FIGO stage and age at cancer diagnosis. **(A)** Changes in unilateral and bilateral oophorectomy rate of patients with ovarian cancer of all stage by age at cancer diagnosis. **(B)** Changes in unilateral and bilateral oophorectomy rate of patients with ovarian cancer by FIGO stage. **(C)** Changes in unilateral and bilateral oophorectomy rate of patients aged 15-49 years with ovarian cancer by FIGO stage. **(D)** Changes in unilateral and bilateral oophorectomy rate of patients aged 50+ years with ovarian cancer by FIGO stage.

Of the patients, 95.5% (N = 27,197) had undergone surgical operations, among whom 7.9%, 50.3%, and 41.8% underwent unilateral oophorectomy, bilateral oophorectomy, and other surgical procedures, respectively (**Table S1**). Patients who underwent unilateral oophorectomy were younger. Most patients who underwent unilateral oophorectomy (70.9%) were younger than 60. Of the patients aged 15–39 years, 33.7% and 22.7% underwent unilateral oophorectomy and bilateral oophorectomy, respectively (**Table 1**). Moreover, there was a decreasing trend in the unilateral oophorectomy rate by age at cancer diagnosis (**Figure 1A**). Similarly, the unilateral oophorectomy rate decreased by FIGO stage (**Figure 1B**), especially for younger patients (**Figure 1C**). Most tumors treated by unilateral oophorectomy were in the Ia stage (53.1%), followed by Ic stage (19.4%). The Hispanic population had a higher unilateral oophorectomy rate (11.3%) than the non-Hispanic population (7.0%) (**Table 1**).

### Survival Analysis of Surgical Interventions for Patients With Ovarian Cancer

**Figure 2:**
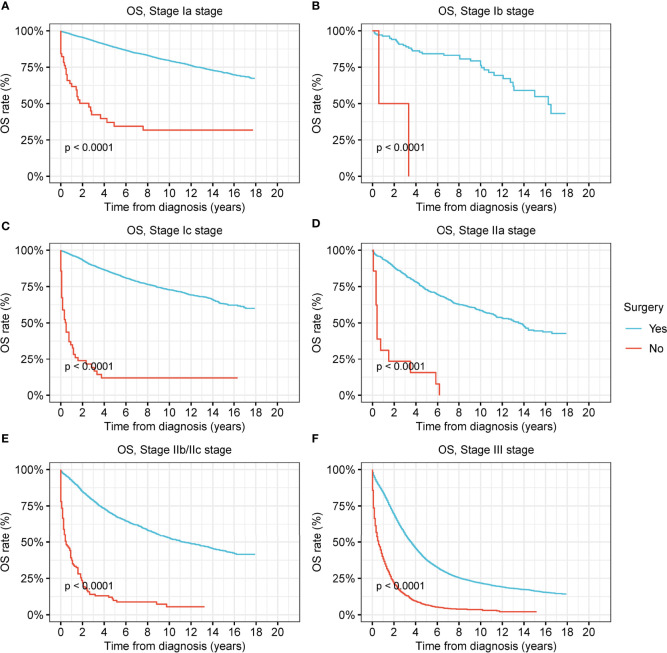
Overall survival (OS) of patients with ovarian cancer by surgery. **(A)** OS of patients with stage-Ia ovarian cancer by surgery. **(B)** OS of patients with stage-Ib ovarian cancer by surgery. **(C)** OS of patients with stage-Ic ovarian cancer by surgery. **(D)** OS of patients with stage-IIa ovarian cancer by surgery. **(E)** OS of patients with stage-IIb/IIc ovarian cancer by surgery. **(F)** OS of patients with stage-III ovarian cancer by surgery.

The OS and DSS of patients who had undergone surgery were significantly better than those of patients who did not (all p < 0.001) (**Figure 2** and **Figure S2**). The prognostic superiority of surgical operation could be observed in ovarian cancer at all stages (**Figures 2B, C** and **Figure S2**).

**Figure 3:**
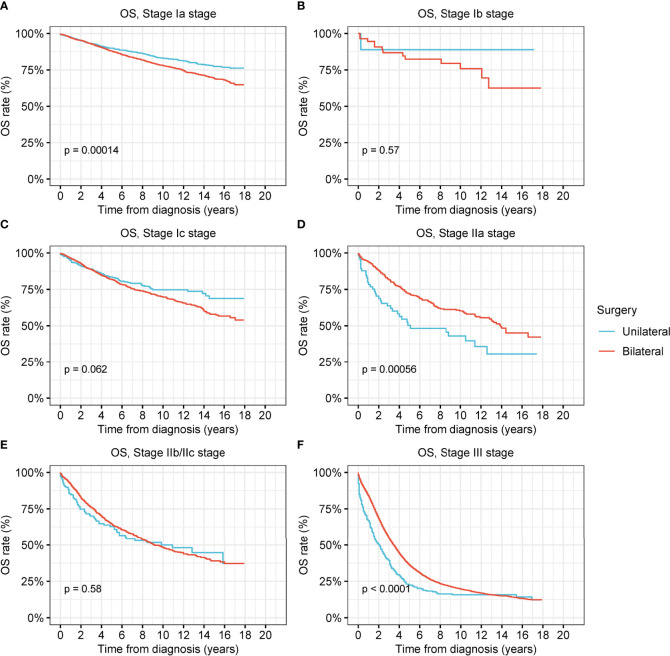
Overall survival (OS) of patients with ovarian cancer by cancer stage and different types of surgical operation. **(A)** OS of patients with stage-Ia ovarian cancer by different types of surgical operation. **(B)** OS of patients with stage-Ib ovarian cancer by different types of surgical operation. **(C)** OS of patients with stage-Ic ovarian cancer by different types of surgical operation. **(D)** OS of patients with stage-IIa ovarian cancer by different types of surgical operation. **(E)** OS of patients with stage-IIb/IIc ovarian cancer by different types of surgical operation. **(F)** OS of patients with stage-III ovarian cancer by different types of surgical operation.

To examine the therapeutic effects of unilateral oophorectomy, we performed survival analyses according to the type of surgical intervention (**Figure 3** and **Figure S3**). In stage-Ia tumor, unilateral oophorectomy was associated with remarkably better OS and DSS compared with bilateral oophorectomy, with a 5-year OS rate of 89.9% for unilateral oophorectomy and 87.9% for bilateral oophorectomy (OS: p < 0.001; DSS: p = 0.01) (**Figure 3A** and **Figure S3A**). For stage-Ib and stage-Ic ovarian tumor, there was no significant difference between the OS and DSS of patients treated by unilateral oophorectomy and those of patients treated by bilateral oophorectomy (**stage Ib**: OS: p = 0.6; DSS: p = 0.8; **stage Ic**: OS: p = 0.06; DSS: p = 0.2) (**Figures 3B, C** and **Figures S3B, C**). For stage-IIa tumors, the OS and DSS after unilateral oophorectomy were significantly worse than those after bilateral oophorectomy (5-year OS: 50.3% vs. 72.0%, p < 0.001; 5-year DSS: 61.6% vs. 72.0%, p < 0.001) (**Figure 3D** and **Figure S3D**). For stage-IIb/IIc tumors, there was no significant difference between the OS and DSS of patients treated by unilateral oophorectomy and those of patients treated by bilateral oophorectomy (OS: p = 0.6; DSS: p = 0.6) (**Figure 3E** and **Figure S3E**). For stage-III tumors, the OS and DSS after unilateral oophorectomy were significantly worse than those after bilateral oophorectomy (OS: p < 0.001; DSS: p < 0.001) (**Figure 3F** and **Figure S3F**).

**Figure 4:**
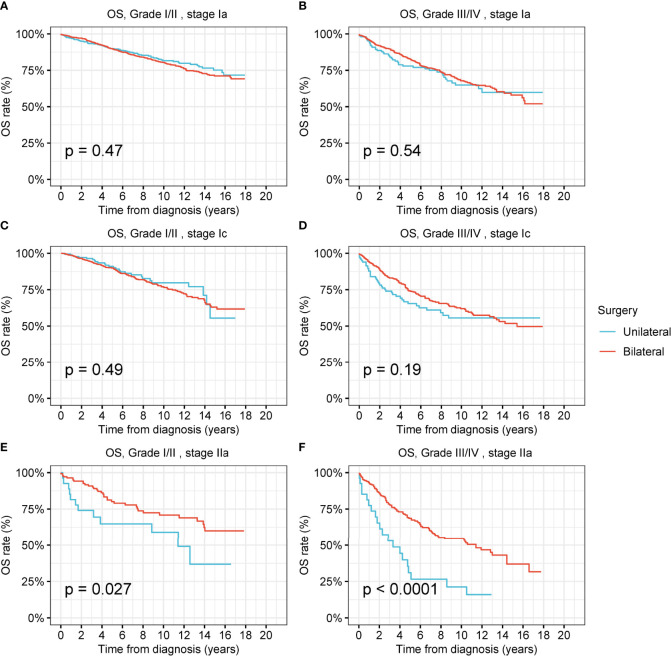
Overall survival (OS) of patients with ovarian cancer by cancer stage, cancer grade and different types of surgical operation. **(A)** OS of patients with low-grade stage-Ia ovarian cancer by different types of surgical operation. **(B)** OS of patients with high-grade stage-Ia ovarian cancer by different types of surgical operation. **(C)** OS of patients with low-grade stage-Ic ovarian cancer by different types of surgical operation. **(D)** OS of patients with high-grade stage-Ic ovarian cancer by different types of surgical operation. **(E)** OS of patients with low-grade stage-IIa ovarian cancer by different types of surgical operation. **(F)** OS of patients with high-grade stage-IIa ovarian cancer by different types of surgical operation.

For low-grade and high-grade stage-I ovarian tumors, there was no significant difference between the OS and DSS of patients treated by unilateral oophorectomy and those of patients treated by bilateral oophorectomy (**Figures 4A–D** and **Figures S4B, C**). The OS of patients with low-grade and high-grade stage-IIa ovarian tumors undergoing unilateral oophorectomy were worse than those of patients undergoing bilateral oophorectomy (low-grade: p = 0.03; high-grade: p < 0.001) (**Figures 4E, F**). The DSS of patients with high-grade stage-IIa ovarian tumor undergoing unilateral oophorectomy were worse than those of patients undergoing bilateral oophorectomy (high-grade: p < 0.001) (**Figure S4F**).

We further analyzed the survival after unilateral oophorectomy or bilateral oophorectomy in patients with ovarian cancers of different histology (**Figures S5–S8**). We found that, except for high-grade stage-Ic serous ovarian cancer (Due to the inadequate cases with stage-Ib tumors, stage-Ib tumors were not included for further analyses hereafter), unilateral oophorectomy was comparable to bilateral oophorectomy in low-grade and high-grade stage-I ovarian cancer of any histology, including serous, mucinous, endometrioid, and clear cell carcinoma (**Figures S5–S8**). For high-grade stage-Ic serous ovarian cancer, the OS after unilateral oophorectomy was significantly worse than that after bilateral oophorectomy (p = 0.03) (**Figure S5D**).

### Survival Analysis of Surgical Interventions by Stage and Age at Cancer Diagnosis

**Figure 5:**
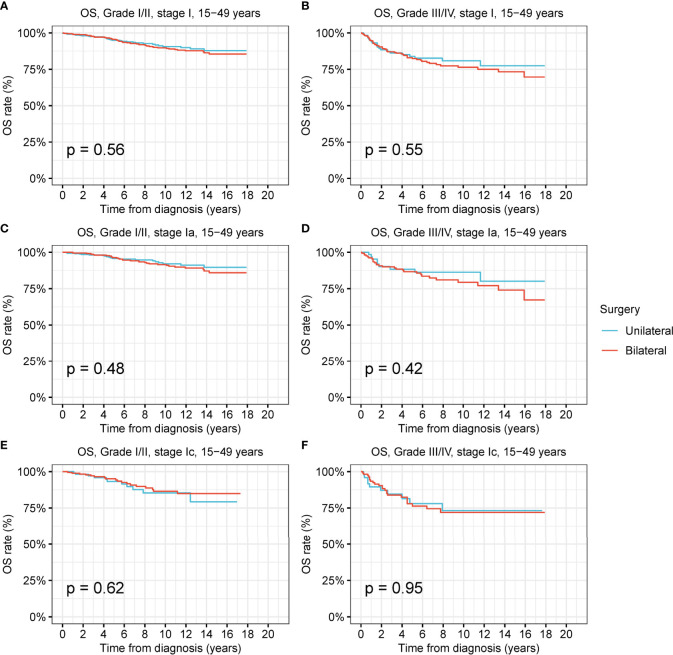
Overall survival (OS) of patients of productive age (15-50 years) with ovarian cancer by cancer stage, cancer grade and different types of surgical operation. **(A)** OS of patients of productive age with low-grade stage-I ovarian cancer by different types of surgical operation. **(B)** OS of patients of productive age with high-grade stage-I ovarian cancer by different types of surgical operation. **(C)** OS of patients of productive age with low-grade stage-Ia ovarian cancer by different types of surgical operation. **(D)** OS of patients of productive age with high-grade stage-Ia ovarian cancer by different types of surgical operation. **(E)** OS of patients of productive age with low-grade stage-Ic ovarian cancer by different types of surgical operation. **(F)** OS of patients of productive age with high-grade stage-Ic ovarian cancer by different types of surgical operation.

**Figure 6:**
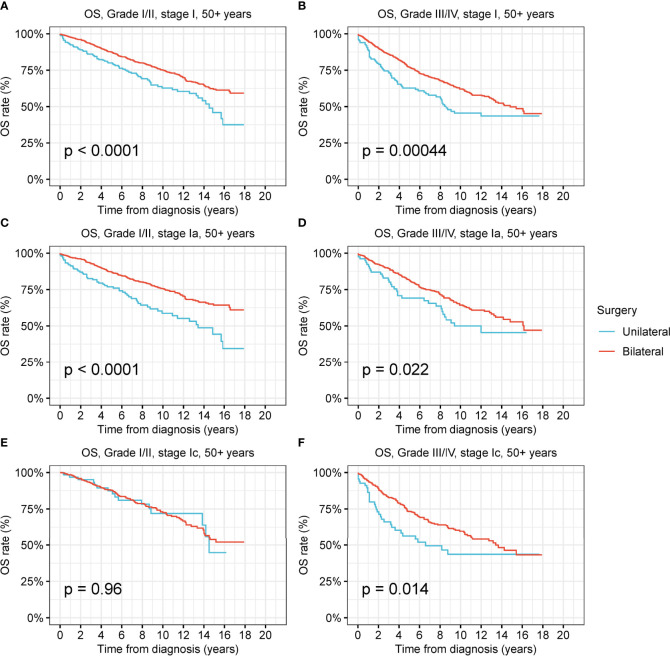
Overall survival (OS) of patients aged 50+ years with ovarian cancer by cancer stage, cancer grade and different types of surgical operation. **(A)** OS of patients aged 50+ years with low-grade stage-I ovarian cancer by different types of surgical operation. **(B)** OS of patients aged 50+ years with high-grade stage-I ovarian cancer by different types of surgical operation. **(C)** OS of patients aged 50+ years with low-grade stage-Ia ovarian cancer by different types of surgical operation. **(D)** OS of patients aged 50+ years with high-grade stage-Ia ovarian cancer by different types of surgical operation. **(E)** OS of patients aged 50+ years with low-grade stage-Ic ovarian cancer by different types of surgical operation. **(F)** OS of patients aged 50+ years with high-grade stage-Ic ovarian cancer by different types of surgical operation.

Female patients of reproductive age had a greater desire to preserve fertility; thus, we investigated the prevalence and therapeutic effects of unilateral oophorectomy in this peculiar population (**Figure 5** and **Figure S9**). Among the reproductive-age women younger than 50 years, 22.8% received unilateral oophorectomy, and 27.9% underwent bilateral oophorectomy. We found that OS and DSS of patients with low-grade and high-grade stage-I receiving unilateral oophorectomy were comparable to those of patients receiving bilateral oophorectomy (all p > 0.05) (**Figure 6** and **Figure S9**). For patients aged 15–59 years with high-grade stage-Ic ovarian tumor, the OS and DSS of patients receiving unilateral oophorectomy were similar to those of patients receiving bilateral oophorectomy (OS: p = 1; DSS: p = 0.7) (**Figure 5F** and **Figure S9F**).

For those aged 50 years and older, the OS of patients with low-grade and high-grade stage-I receiving unilateral oophorectomy was significantly worse than that of patients receiving bilateral oophorectomy (low-grade: p < 0.001; high-grade: p < 0.001) (**Figures 6A, B**). The DSS of patients aged 50 years and older with low-grade stage-I receiving unilateral oophorectomy were similar to those of patients receiving bilateral oophorectomy (p = 0.2) (**Figure S10A**). The DSS of patients aged 50 years and older with high-grade stage-I receiving unilateral oophorectomy were worse than that of patients receiving bilateral oophorectomy (p < 0.001) (**Figure S10B**). For patients aged 50 years and older with low-grade stage-Ia ovarian tumor, the OS and DSS of patients receiving unilateral oophorectomy were significantly worse than those of patients receiving bilateral oophorectomy (OS: p < 0.001; DSS: p = 0.01) (**Figure 6C** and **Figure S10C**).

### Comorbidity Analysis of Patients With Ovarian Cancer Treated by Different Surgical Interventions

**Figure 7:**
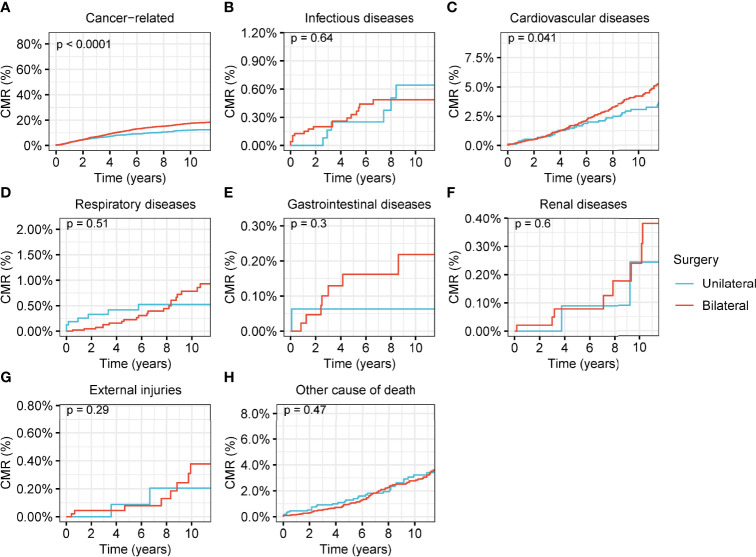
Cumulative mortality rate (CMR) among women of productive age with stage-I ovarian cancer by different types of surgical operation. **(A)** CMR from cancer-related deaths among women of productive age with stage-I ovarian cancer by different types of surgical operation. **(B)** CMR from infectious diseases among women of productive age with stage-I ovarian cancer by different types of surgical operation. **(C)** CMR from cardiovascular diseases among women of productive age with stage-I ovarian cancer by different types of surgical operation. **(D)** CMR from respiratory diseases among women of productive age with stage-I ovarian cancer by different types of surgical operation. **(E)** CMR from gastrointestinal diseases among women of productive age with stage-I ovarian cancer by different types of surgical operation. **(F)** CMR from renal diseases among women of productive age with stage-I ovarian cancer by different types of surgical operation. **(G)** CMR from external injuries among women of productive age with stage-I ovarian cancer by different types of surgical operation. **(H)** CMR from other non-cancer causes among women of productive age with stage-I ovarian cancer by different types of surgical operation.

A comorbidity analysis was carried out on the causes of death for the patients with ovarian cancer **(** **Figure 7**, **Figure S11**, and **Figure S12**). In stage-I ovarian cancer, the CMR of cancer-related deaths was significantly lower in patients who underwent unilateral oophorectomy than in those who underwent bilateral oophorectomy (p < 0.001) (**Figure 7A**). CVDs were also remarkably decreased in patients who underwent unilateral oophorectomy (5-year CMR: unilateral oophorectomy, 1.5%; bilateral oophorectomy, 1.7%; p = 0.04) (**Figure 7C**). For stage-II and stage-III tumors, there were no significant differences between the CMR of unilateral and bilateral oophorectomy for both cancer-related deaths and non-cancer comorbidities (**Figure S11** and **Figure S12**).

## Discussion

In this population-based study involving more than 28,000 women with ovarian cancer, we compared the prevalence and therapeutic efficacy of unilateral oophorectomy with bilateral oophorectomy. We found that unilateral oophorectomy exhibited excellent oncological superiority and was equivalent to bilateral oophorectomy for stage-I ovarian tumors among women of productive age; this equivalence to bilateral oophorectomy remained true for high-grade stage-Ic ovarian tumors. For patients aged 50 years and older, the performance of unilateral oophorectomy was worse than that of bilateral oophorectomy, even for low-grade stage-Ia ovarian tumors. These results indicated that unilateral oophorectomy was valuable for stage-I ovarian tumors among women of productive age.

Unilateral oophorectomy has the advantages of preserving fertility and part or full function of the ovary, while fertility is completely destroyed after bilateral oophorectomy. Fertility preservation is an important component of cervical cancer survivors’ overall quality of life (20). Fertility-preserving procedures in cases of borderline ovarian tumors are now well-established because this type of lesion is often diagnosed in young women whose fertility issues are primordial (21). The status of fertility-preserving procedures in malignant ovarian cancer remains controversial. Data on the conservative management of ovarian cancer are still limited; however, the oncologic safety of fertility-sparing procedures in early ovarian cancer has been confirmed (22–24). Researchers also proposed that high-risk disease should not be considered a contraindication to conservative surgery (23, 25). This procedure is mainly limited to women with IA grade 1 disease who wish to preserve their fertility. For some investigators, fertility-sparing procedures were found to be safe in women with more advanced-stage disease until stage IC (26). Our results further confirmed the potential candidates for this procedure. We found that age is an important factor in selecting potential candidates, as unilateral oophorectomy is valuable for stage-I ovarian tumors among women of productive age, even for high-grade stage-Ic diseases. In contrast, the performance of unilateral oophorectomy is demonstrated to be greatly weakened by age. For patients aged 50 years or older, the long-term survival after unilateral oophorectomy is worse than that after bilateral oophorectomy, even for the tumors with the lowest risk, namely, the low-grade stage-Ia tumors. Therefore, we recommend the inclusion criteria of unilateral oophorectomy be extended to high-grade stage-Ic diseases; in contrast, for those aged 50 years and older without fertility desire, unilateral oophorectomy is not recommended, and bilateral oophorectomy should be adopted as the first choice.

The major limitations of this procedure are the underlying risks of residual tumor, tumor recurrence, and possible newly occurring carcinoma in the remaining ovarian tissue. To address these concerns, postoperative chemotherapy, radiotherapy, or molecular targeted therapy should be employed for the high-risk population (27). Precise diagnosis and stage of the disease before the surgery are vital to guarantee the tumor clearance of surgery (28). Minimally invasive surgery, if necessary, is a viable approach to accurately diagnose and stage the tumor (29–31). Routine screening and active follow-up should be performed after this procedure to avoid future rumor recurrence or newly developed tumors.

Our results revealed that unilateral oophorectomy can decrease the long-term mortality risk of CVD; this might be a consequence of the stable hormone levels generated from the preservation of part or full ovarian function, while bilateral oophorectomy will lead to a sudden disruption in the secretion of sex hormones, mainly estrogen. In addition to its powerful roles in regulating the development and homeostasis of reproductive tissues, estrogen provides critical signaling and trophic support to a range of tissues throughout the body and across the lifespan through the activation of estrogen receptors, ERα (encoded by ESR1), ERβ (encoded by ESR2), and G-protein-coupled estrogen receptor (GPER; also known as GPR30) (32–35). Estrogens act in target tissues through estrogen receptors and G protein-coupled ERα to reduce CVD risk (33). Premenopausal women are protected from CVD relative to age-matched men (36, 37), and low levels of estrogens (i.e., hypo-estrogenemia) in young women (18–40 years) increase CVD risk (38). Moreover, early menopause (before 40 years of age) (39) is associated with accelerated atherosclerosis, a 2.6-fold increase in CVD risk (40), and an increased risk of CVD-related mortality (41, 42). These studies supported the role of estrogens in determining CVD risk. Therefore, after bilateral oophorectomy, the destruction of ovarian function results in the demand for hormone replacement therapy (HRT), while unilateral oophorectomy, which maintains part or whole of ovarian function, does not need HRT. Furthermore, HRT may be difficult and even dangerous for some women. Endogenous estrogen from the remaining ovary after unilateral oophorectomy eliminates these difficulties and dangers.

This study had several limitations. First, given the study’s descriptive and retrospective design, we could not prospectively assess the effects of surgical interventions in patients with ovarian cancer and could not draw causal inferences. Second, we could not assess the patients’ physical conditions, comorbidities, and other health factors. Given the high incidence of comorbidities, cognitive impairment, frailty, functional losses, social isolation, and other factors in this population, it is important to assess these variables when proposing treatment decisions; however, the SEER program did not provide this information. Third, we could not investigate the influence of other therapies, such as radiotherapy or chemotherapy. The SEER program only provided detailed information on surgical operations.

Notwithstanding these limitations, this study may contribute to the surgical interventions and cancer surveillance literature for ovarian cancer. The strength of this study is that the data were derived from a high-quality, population-based, real-world cancer registry. Real-world data reflect the realistic effects of different interventions in the real scenario of cancer treatment, which may avoid the limitations of clinical trials. The implications of this study are important for the development of ovarian cancer.

## Conclusions

In conclusion, unilateral oophorectomy exhibited excellent oncological superiority and was equivalent to bilateral oophorectomy for stage-I ovarian tumors among women of productive age. For women of reproductive age, the criteria for unilateral oophorectomy can be appropriately broadened to high-grade stage-Ic diseases because of the comparable performance of unilateral oophorectomy in this population. For those aged 50 years and older without fertility desire, unilateral oophorectomy is not recommended, and bilateral oophorectomy should be adopted as the first choice. Moreover, unilateral oophorectomy can reduce mortality and CVD risk in women. As unilateral oophorectomy has the advantage of preserving fertility and the hormone secretion function of the ovary, guidance on selecting appropriate candidates should be developed.

## Data Availability Statement

Publicly available datasets were analyzed in this study. This data can be found here: http://www.seer.cancer.gov.

## Author Contributions

WZ and KW designed the study. JX, YL, and GF performed the research. ZZ, KW, and WZ developed the typescript. All of the authors approved the typescript and agree to submit for publication.

## Funding

This study was supported by National Key Research and Development Program of China (2021YFC2009105) and National Natural Science Foundation of China (82002760).

## Conflict of Interest

The authors declare that the research was conducted in the absence of any commercial or financial relationships that could be construed as a potential conflict of interest.

## Publisher’s Note

All claims expressed in this article are solely those of the authors and do not necessarily represent those of their affiliated organizations, or those of the publisher, the editors and the reviewers. Any product that may be evaluated in this article, or claim that may be made by its manufacturer, is not guaranteed or endorsed by the publisher.
