# Supplementary material for: Prevalence and Outcomes of Unilateral Versus Bilateral Oophorectomy in Women With Ovarian Cancer: A Population-Based Study
Source: Front Oncol. 2022 Jul 8;12:866443. doi: 10.3389/fonc.2022.866443 (PMC9304749; doi:10.3389/fonc.2022.866443)
Supplement: Supplementary file 1 [file DataSheet_1.docx]

**Supplementary Tables and Figures**

**Table S1** Proportion of patients underwent different types of surgical interventions.

| Surgery | No. of patients (%) | No. of deaths (%) |
| --- | --- | --- |
| All | 28,480 (100%) | 11,517 (100%) |
| Surgery | 27,197 (95.5%) | 10,424 (90.5%) |
| Unilateral | 2,145 (7.5%) | 598 (5.2%) |
| Bilateral | 13,678 (48%) | 6,205 (53.9%) |
| Other surgery | 11,374 (39.9%) | 3,621 (31.4%) |
| No surgery | 1,283 (4.5%) | 1,093 (9.5%) |


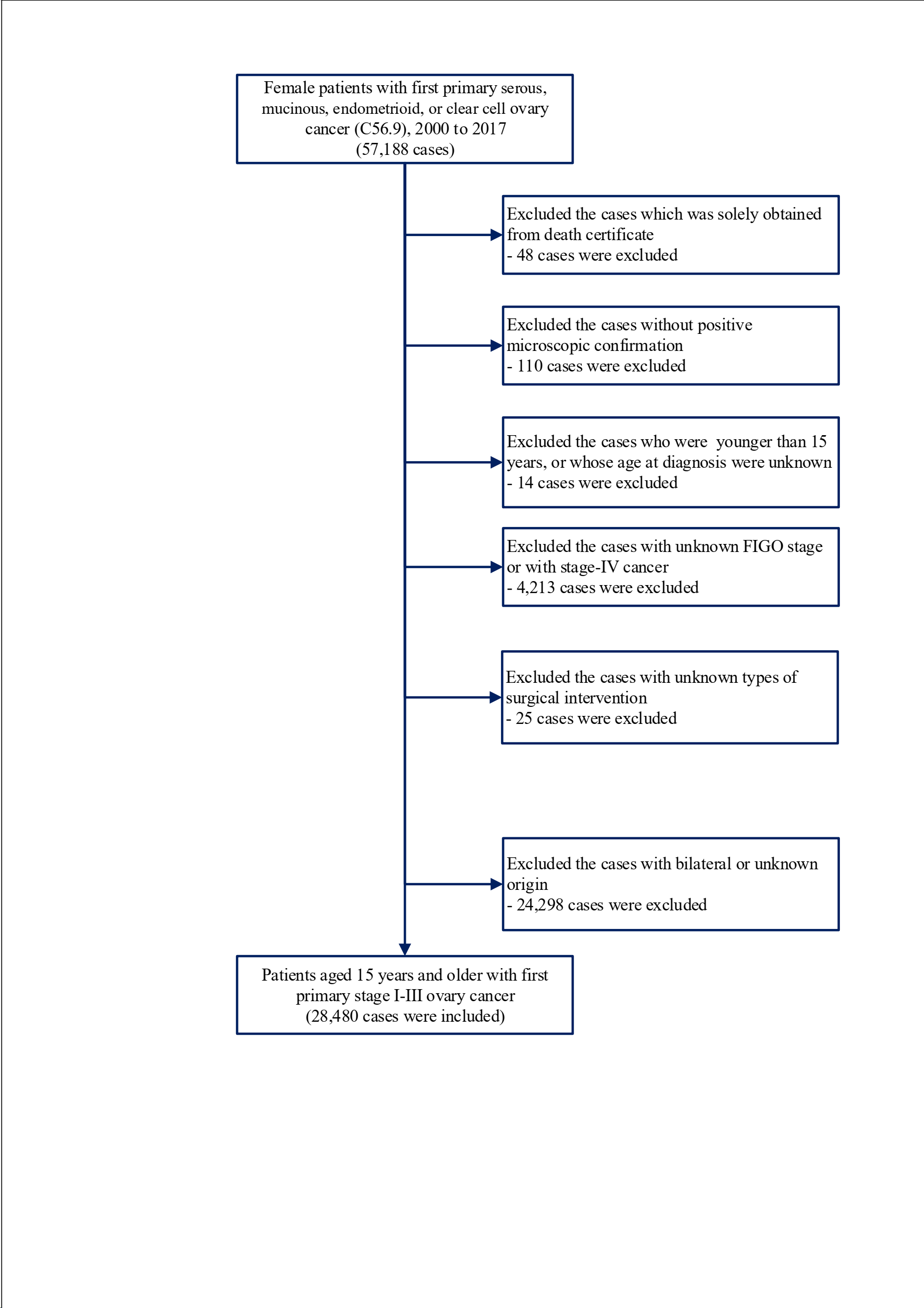


**Figure S1** Inclusion and exclusion criteria of patients included in this study.


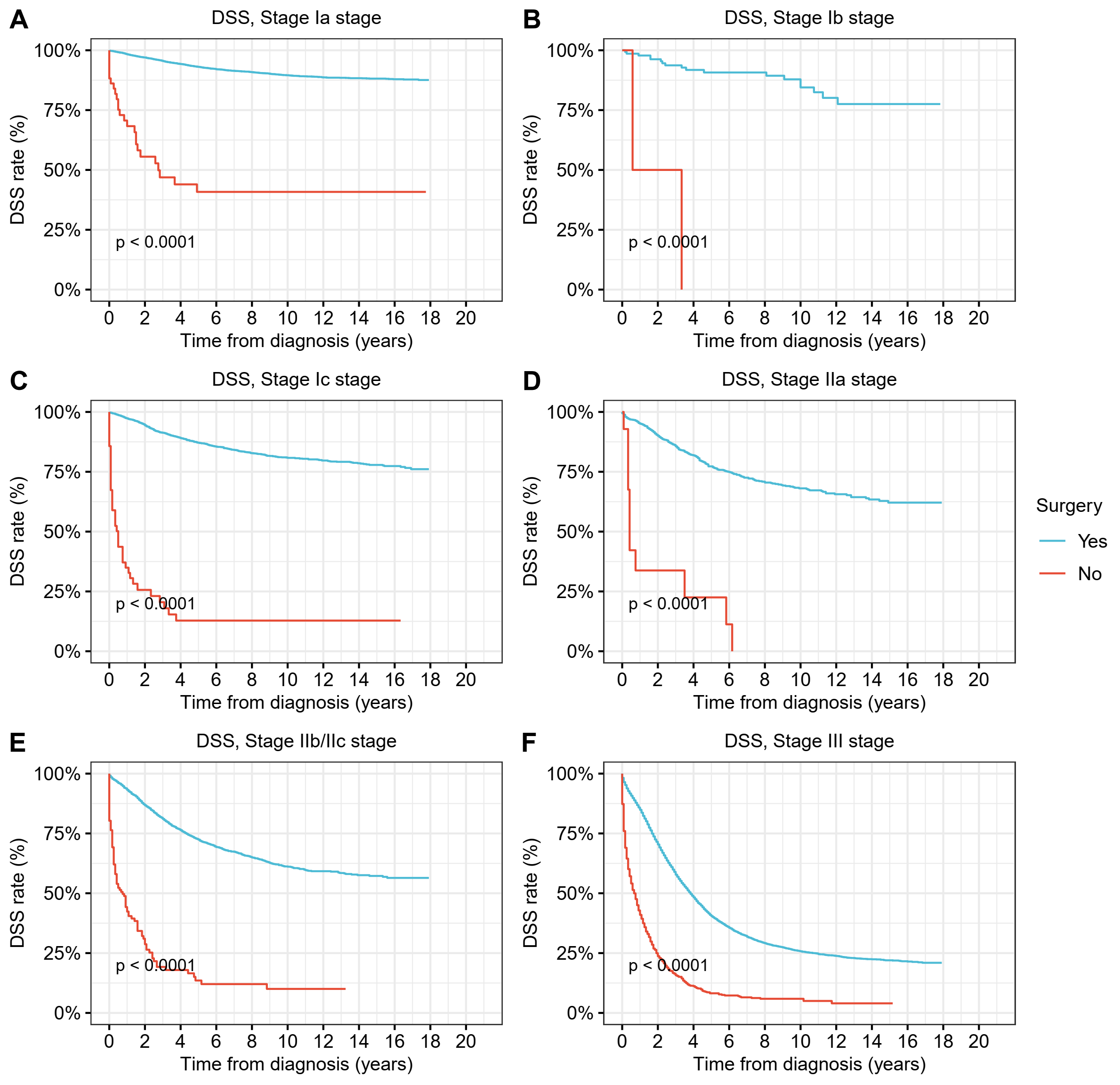


**Figure S2.** Disease-specific survival (DSS) of patients with ovarian cancer by surgery. (A) DSS of patients with stage-Ia ovarian cancer by surgery. (B) DSS of patients with stage-Ib ovarian cancer by surgery. (C) DSS of patients with stage-Ic ovarian cancer by surgery. (D) DSS of patients with stage-IIa ovarian cancer by surgery. (E) DSS of patients with stage-IIb/IIc ovarian cancer by surgery. (F) DSS of patients with stage-III ovarian cancer by surgery.


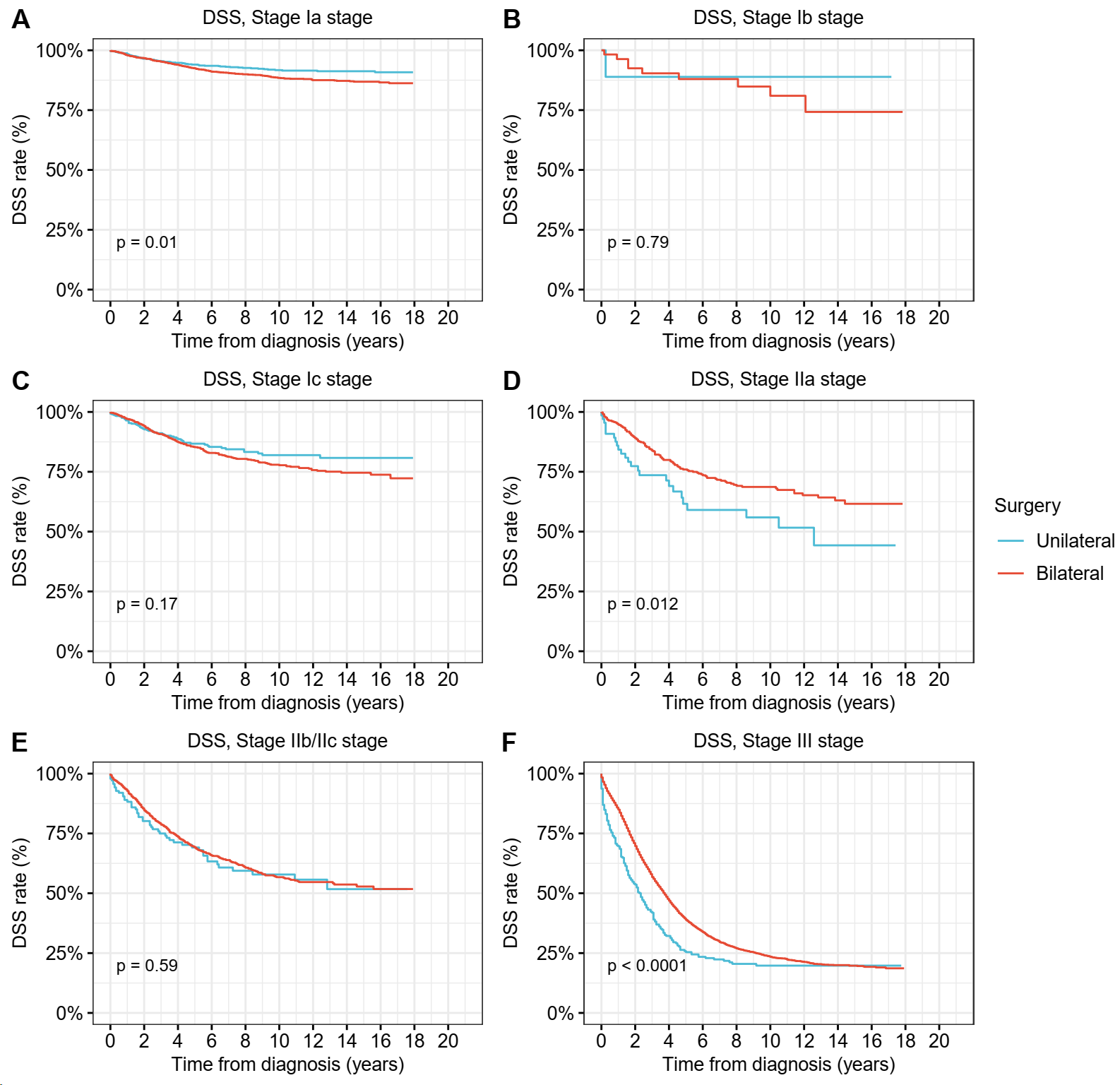


**Figure S3.** Disease-specific survival (DSS) of patients with ovarian cancer by cancer stage and different types of surgical operation. (A) DSS of patients with stage-Ia ovarian cancer by different types of surgical operation. (B) DSS of patients with stage-Ib ovarian cancer by different types of surgical operation. (C) DSS of patients with stage-Ic ovarian cancer by different types of surgical operation. (D) DSS of patients with stage-IIa ovarian cancer by different types of surgical operation. (E) DSS of patients with stage-IIb/IIc ovarian cancer by different types of surgical operation. (F) DSS of patients with stage-III ovarian cancer by different types of surgical operation.


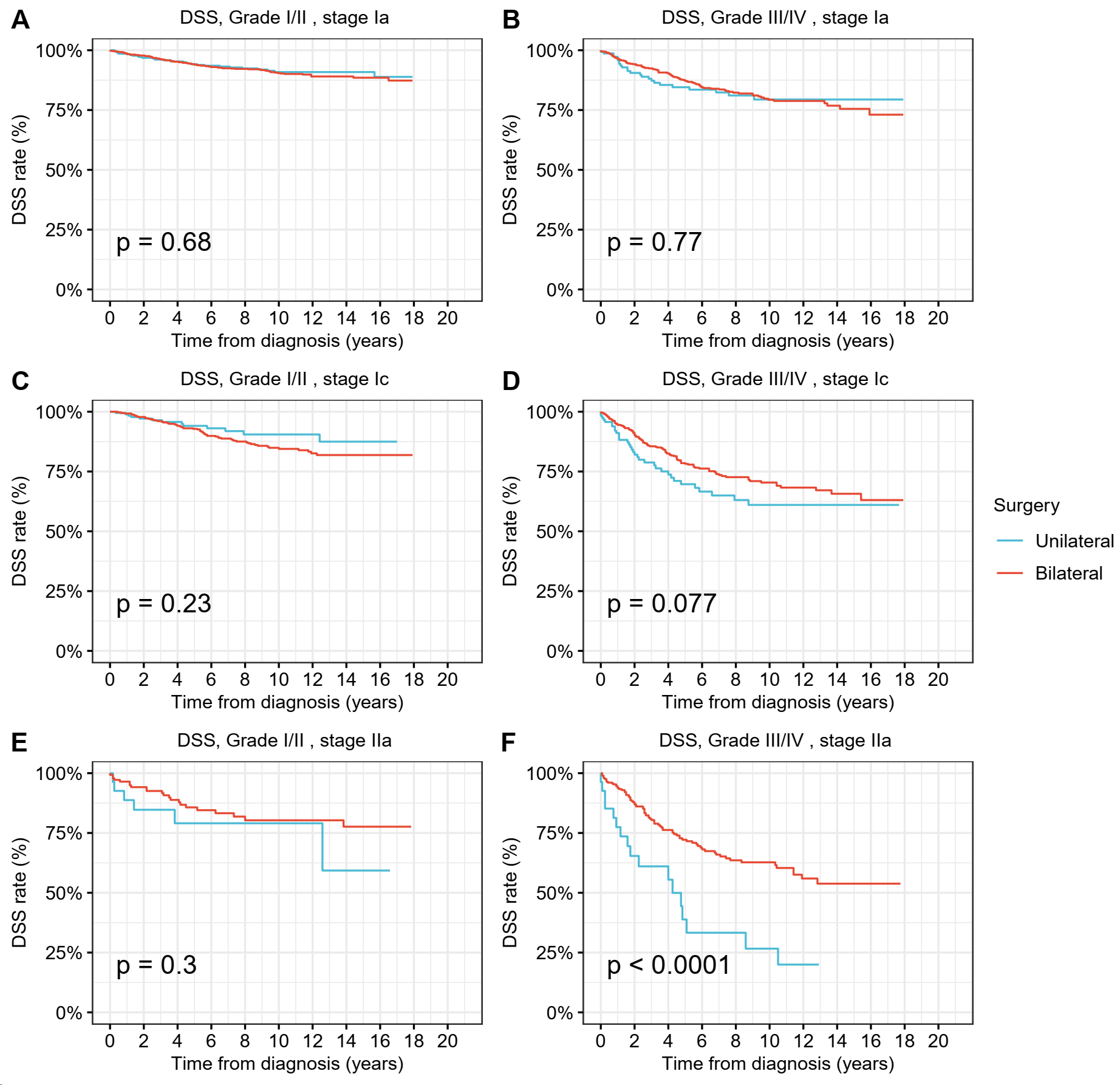


**Figure S4.** Disease-specific survival (DSS) of patients with ovarian cancer by cancer stage, cancer grade and different types of surgical operation. (A) DSS of patients with low-grade stage-Ia ovarian cancer by different types of surgical operation. (B) DSS of patients with high-grade stage-Ia ovarian cancer by different types of surgical operation. (C) DSS of patients with low-grade stage-Ic ovarian cancer by different types of surgical operation. (D) DSS of patients with high-grade stage-Ic ovarian cancer by different types of surgical operation. (E) DSS of patients with low-grade stage-IIa ovarian cancer by different types of surgical operation. (F) DSS of patients with high-grade stage-IIa ovarian cancer by different types of surgical operation.


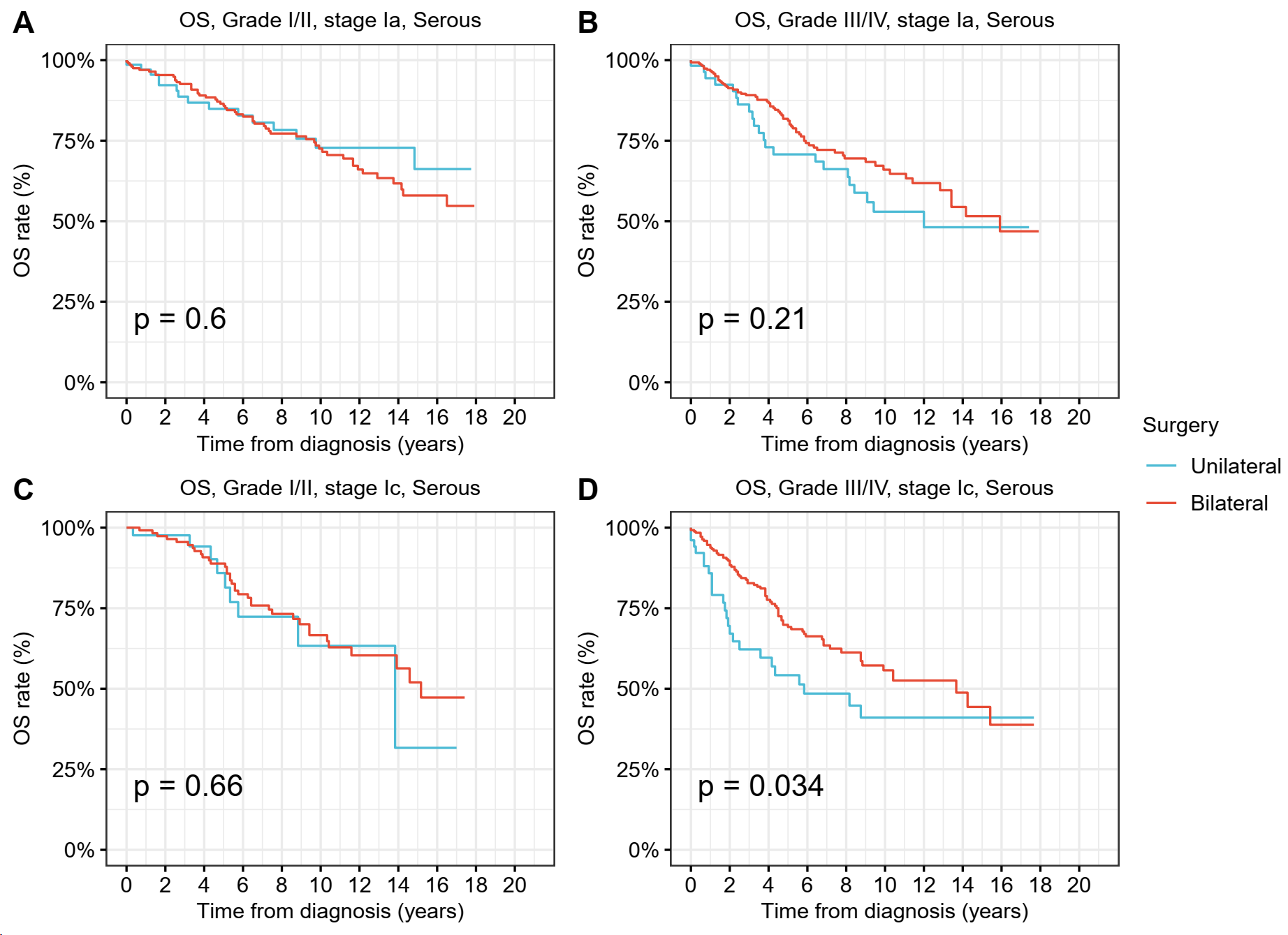


**Figure S5.** Disease-specific survival (DSS) of patients with serous ovarian cancer by cancer stage, cancer grade and different types of surgical operation. (A) DSS of patients with low-grade stage-Ia serous ovarian cancer by different types of surgical operation. (B) DSS of patients with high-grade stage-Ia serous ovarian cancer by different types of surgical operation. (C) DSS of patients with low-grade stage-Ic serous ovarian cancer by different types of surgical operation. (D) DSS of patients with high-grade stage-Ic serous ovarian cancer by different types of surgical operation.


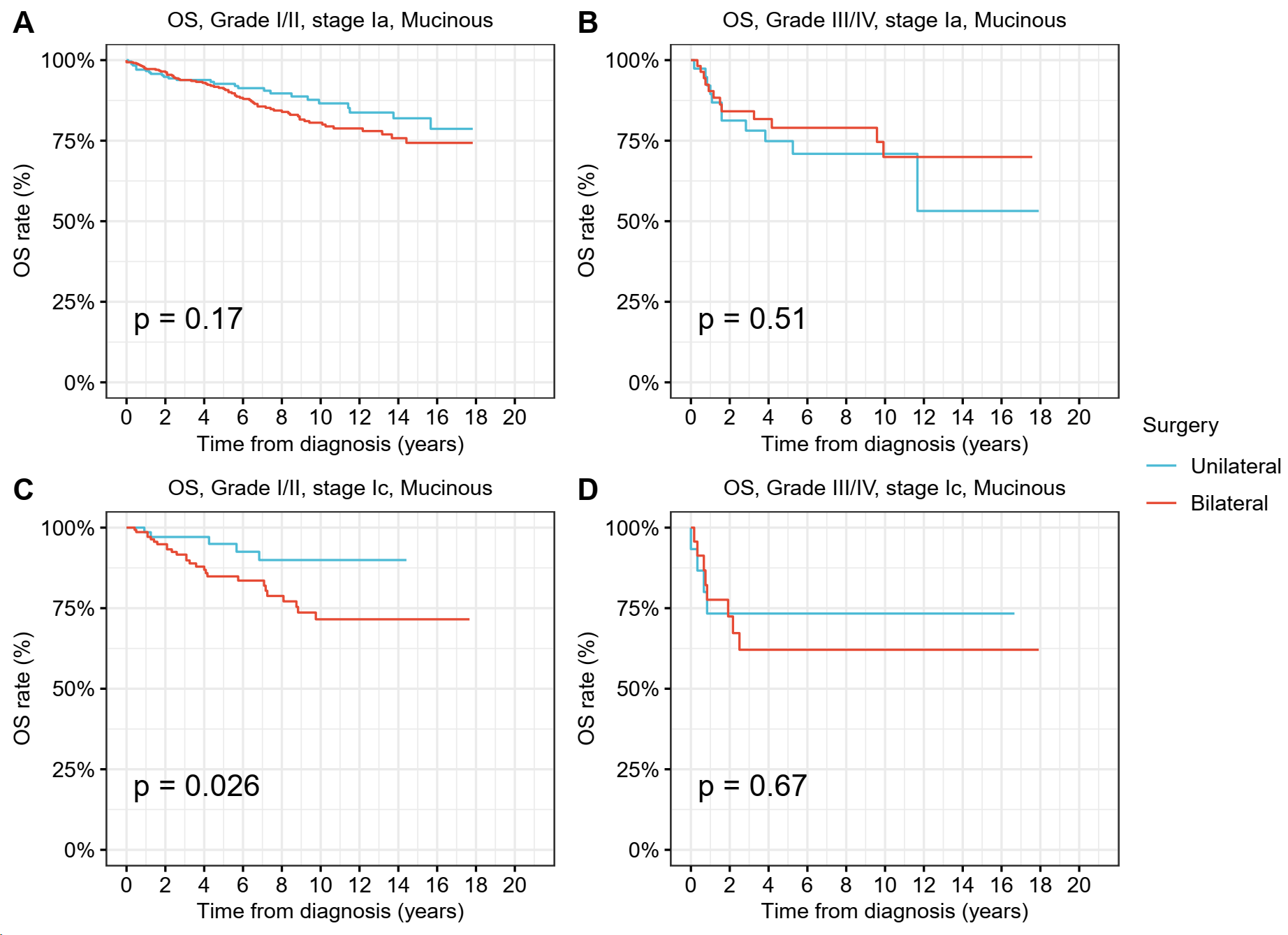


**Figure S6.** Disease-specific survival (DSS) of patients with mucinous ovarian cancer by cancer stage, cancer grade and different types of surgical operation. (A) DSS of patients with low-grade stage-Ia mucinous ovarian cancer by different types of surgical operation. (B) DSS of patients with high-grade stage-Ia mucinous ovarian cancer by different types of surgical operation. (C) DSS of patients with low-grade stage-Ic mucinous ovarian cancer by different types of surgical operation. (D) DSS of patients with high-grade stage-Ic mucinous ovarian cancer by different types of surgical operation.


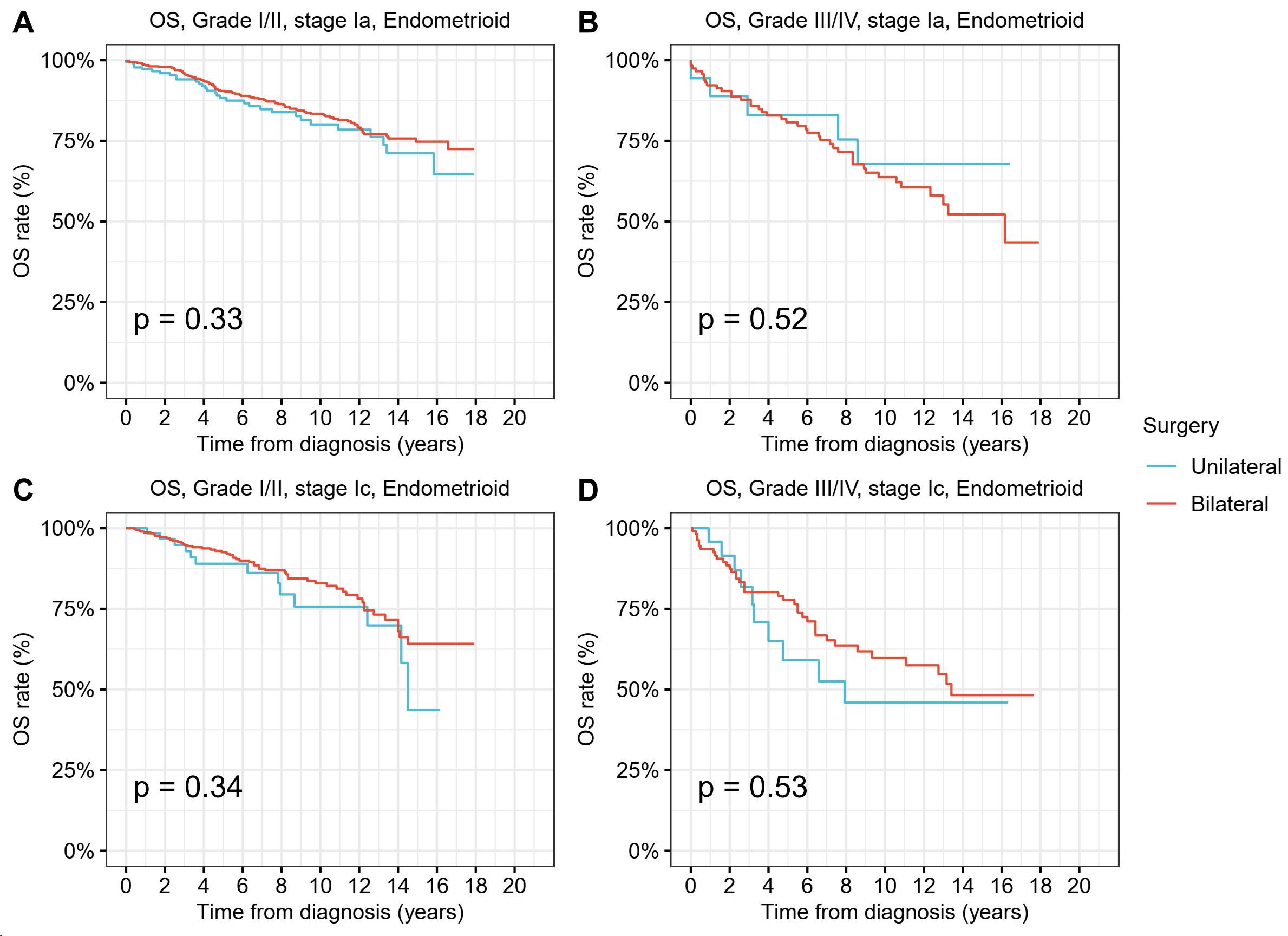


**Figure S7.** Disease-specific survival (DSS) of patients with endometrioid ovarian cancer by cancer stage, cancer grade and different types of surgical operation. (A) DSS of patients with low-grade stage-Ia endometrioid ovarian cancer by different types of surgical operation. (B) DSS of patients with high-grade stage-Ia endometrioid ovarian cancer by different types of surgical operation. (C) DSS of patients with low-grade stage-Ic endometrioid ovarian cancer by different types of surgical operation. (D) DSS of patients with high-grade stage-Ic endometrioid ovarian cancer by different types of surgical operation.


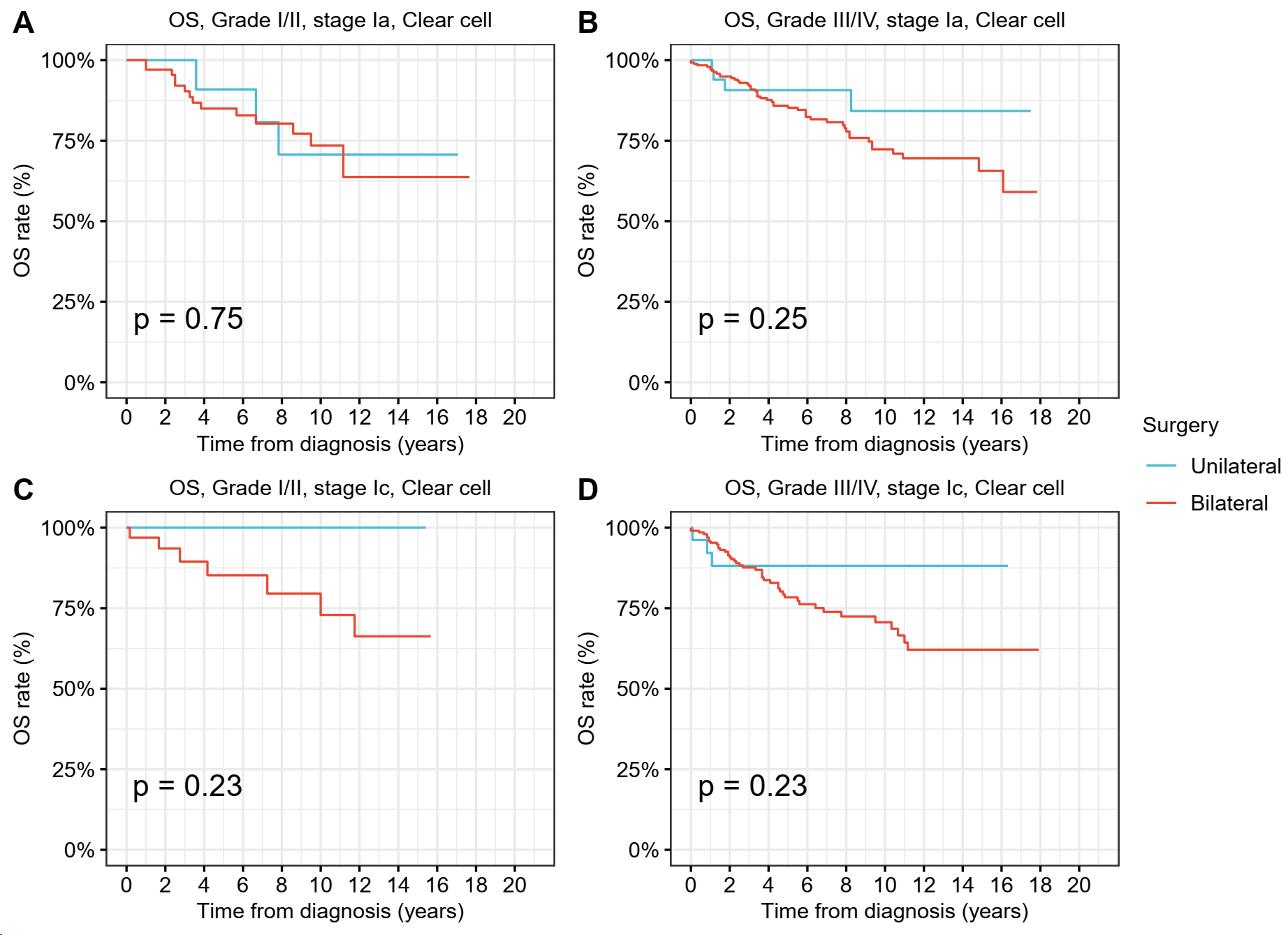


**Figure S8.** Disease-specific survival (DSS) of patients with clear cell ovarian cancer by cancer stage, cancer grade and different types of surgical operation. (A) DSS of patients with low-grade stage-Ia clear cell ovarian cancer by different types of surgical operation. (B) DSS of patients with high-grade stage-Ia clear cell ovarian cancer by different types of surgical operation. (C) DSS of patients with low-grade stage-Ic clear cell ovarian cancer by different types of surgical operation. (D) DSS of patients with high-grade stage-Ic clear cell ovarian cancer by different types of surgical operation.


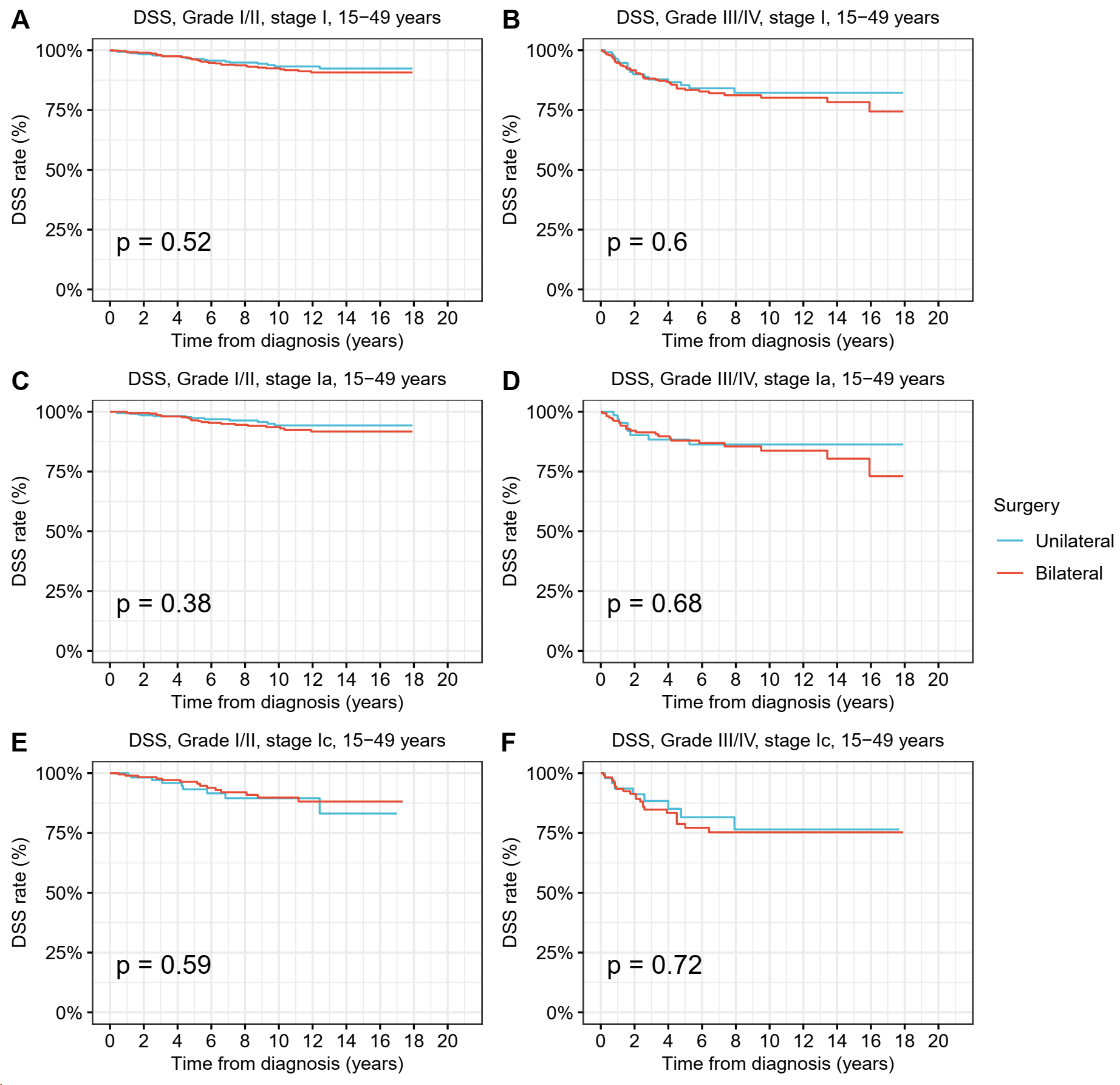


**Figure S9.** Disease-specific survival (DSS) of patients of productive age (15-50 years) with ovarian cancer by cancer stage, cancer grade and different types of surgical operation. (A) DSS of patients of productive age with low-grade stage-I ovarian cancer by different types of surgical operation. (B) DSS of patients of productive age with high-grade stage-I ovarian cancer by different types of surgical operation. (C) DSS of patients of productive age with low-grade stage-Ia ovarian cancer by different types of surgical operation. (D) DSS of patients of productive age with high-grade stage-Ia ovarian cancer by different types of surgical operation. (E) DSS of patients of productive age with low-grade stage-Ic ovarian cancer by different types of surgical operation. (F) DSS of patients of productive age with high-grade stage-Ic ovarian cancer by different types of surgical operation.


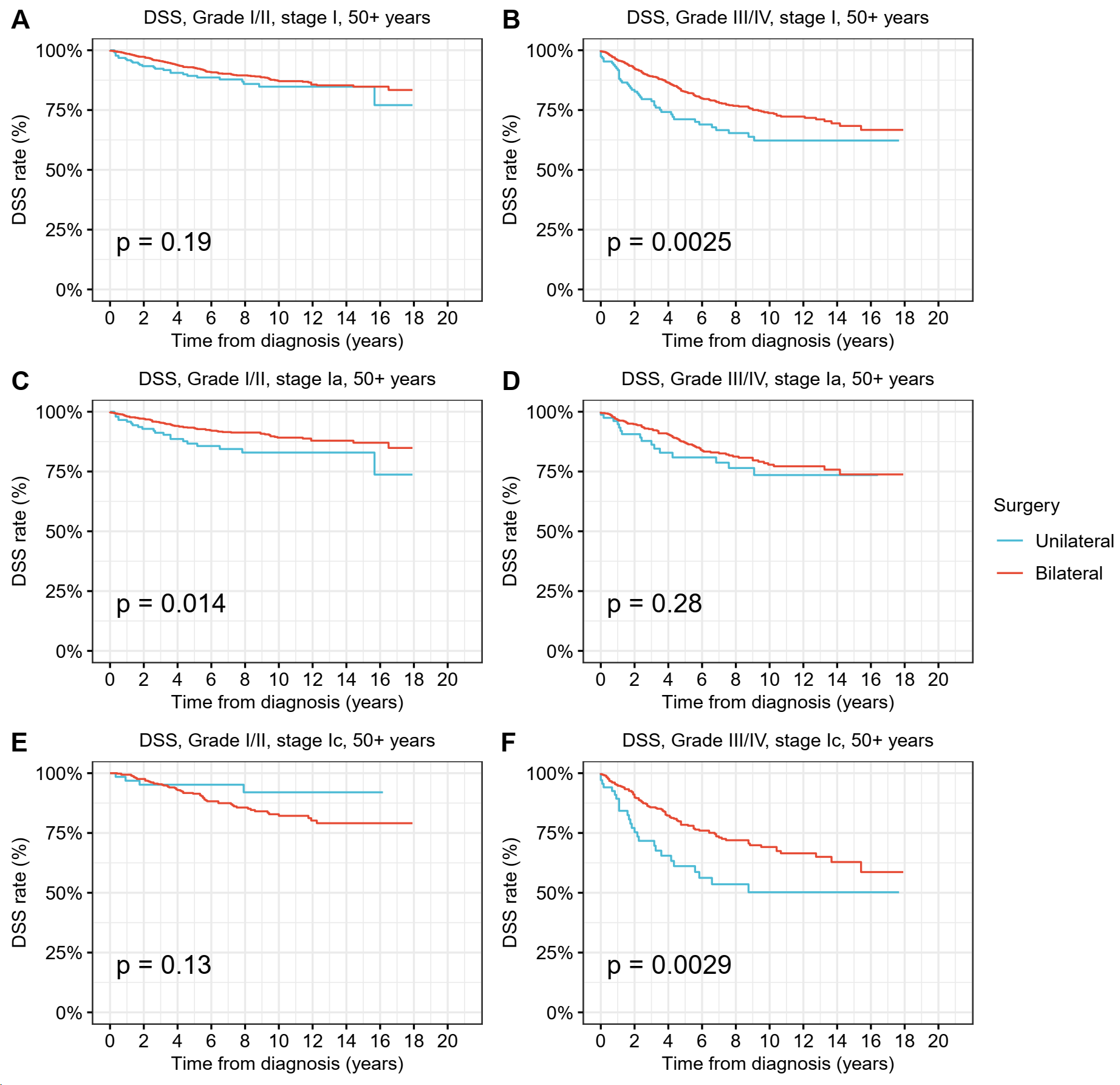


**Figure S10.** Disease-specific survival (DSS) of patients aged 50+ years with ovarian cancer by cancer stage, cancer grade and different types of surgical operation. (A) DSS of patients aged 50+ years with low-grade stage-I ovarian cancer by different types of surgical operation. (B) DSS of patients aged 50+ years with high-grade stage-I ovarian cancer by different types of surgical operation. (C) DSS of patients aged 50+ years with low-grade stage-Ia ovarian cancer by different types of surgical operation. (D) DSS of patients aged 50+ years with high-grade stage-Ia ovarian cancer by different types of surgical operation. (E) DSS of patients aged 50+ years with low-grade stage-Ic ovarian cancer by different types of surgical operation. (F) DSS of patients aged 50+ years with high-grade stage-Ic ovarian cancer by different types of surgical operation.


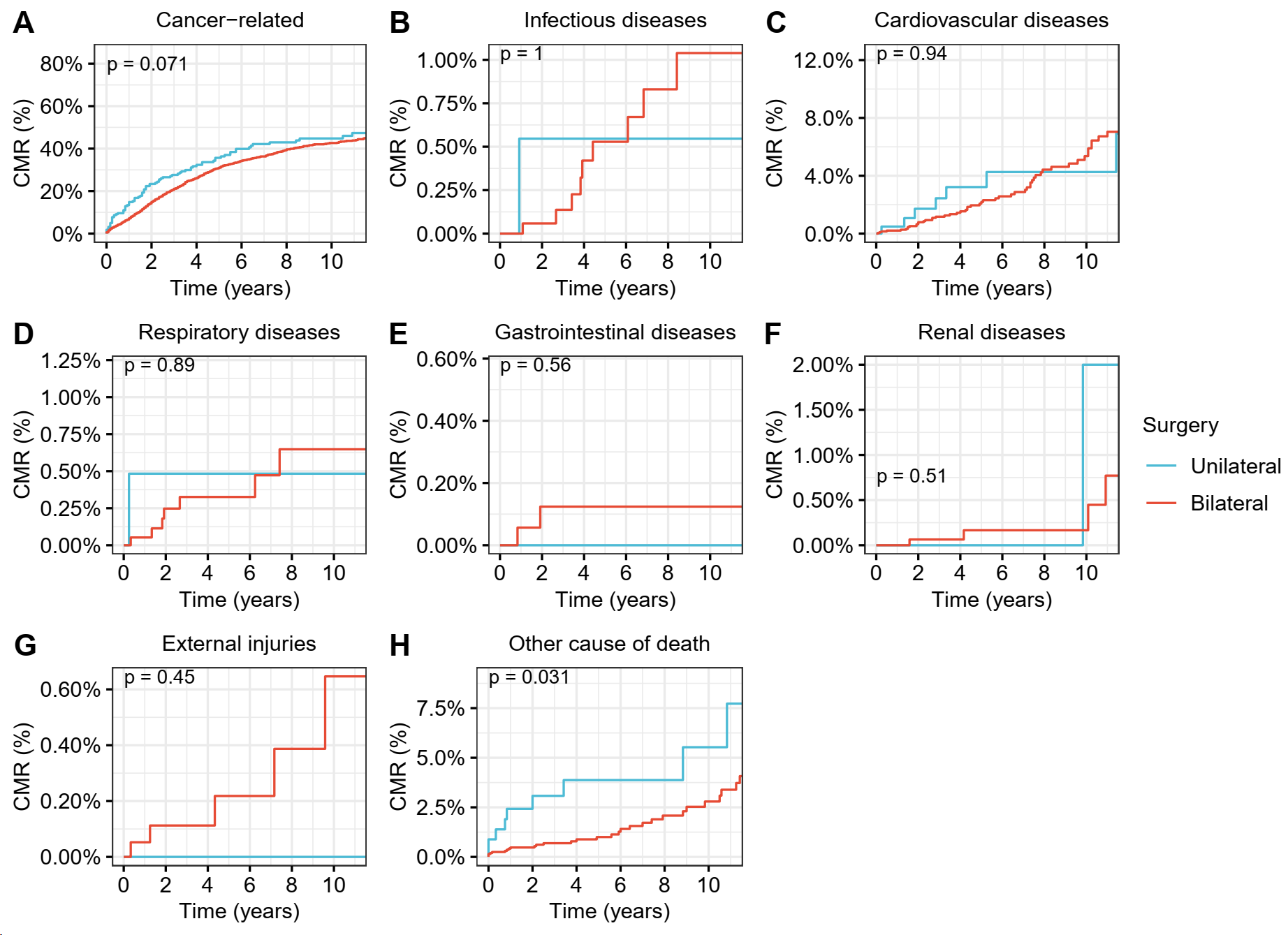


**Figure S11.** Cumulative mortality rate (CMR) among women of productive age with stage-II ovarian cancer by different types of surgical operation. (A) CMR from cancer-related deaths among women of productive age with stage-II ovarian cancer by different types of surgical operation. (B) CMR from infectious diseases among women of productive age with stage-II ovarian cancer by different types of surgical operation. (C) CMR from cardiovascular diseases among women of productive age with stage-II ovarian cancer by different types of surgical operation. (D) CMR from respiratory diseases among women of productive age with stage-II ovarian cancer by different types of surgical operation. (E) CMR from gastrointestinal diseases among women of productive age with stage-II ovarian cancer by different types of surgical operation. (F) CMR from renal diseases among women of productive age with stage-II ovarian cancer by different types of surgical operation. (G) CMR from external injuries among women of productive age with stage-II ovarian cancer by different types of surgical operation. (H) CMR from other non-cancer causes among women of productive age with stage-II ovarian cancer by different types of surgical operation.


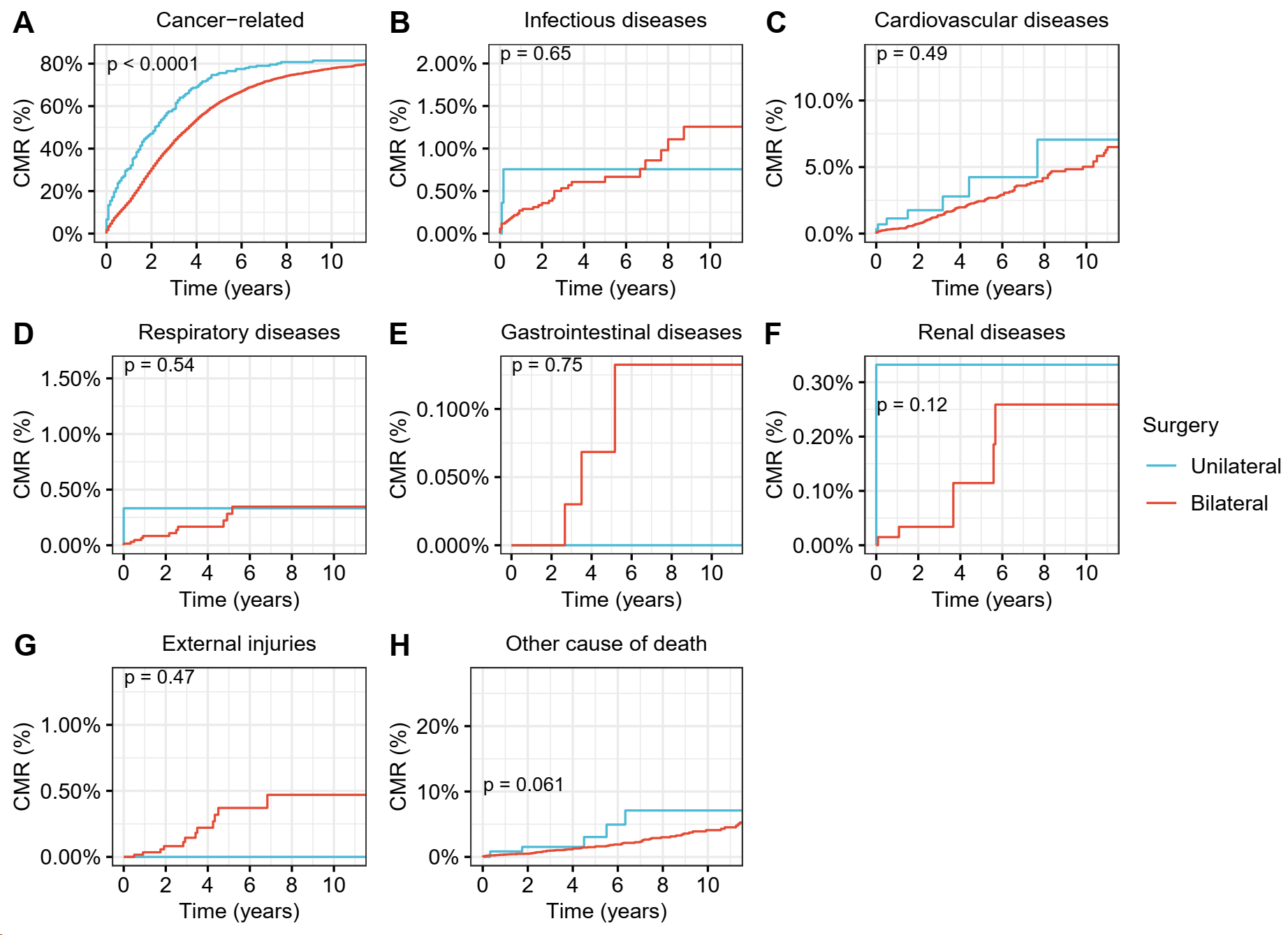


**Figure S12.** Cumulative mortality rate (CMR) among women of productive age with stage-III ovarian cancer by different types of surgical operation. (A) CMR from cancer-related deaths among women of productive age with stage-III ovarian cancer by different types of surgical operation. (B) CMR from infectious diseases among women of productive age with stage-III ovarian cancer by different types of surgical operation. (C) CMR from cardiovascular diseases among women of productive age with stage-III ovarian cancer by different types of surgical operation. (D) CMR from respiratory diseases among women of productive age with stage-III ovarian cancer by different types of surgical operation. (E) CMR from gastrointestinal diseases among women of productive age with stage-III ovarian cancer by different types of surgical operation. (F) CMR from renal diseases among women of productive age with stage-III ovarian cancer by different types of surgical operation. (G) CMR from external injuries among women of productive age with stage-III ovarian cancer by different types of surgical operation. (H) CMR from other non-cancer causes among women of productive age with stage-III ovarian cancer by different types of surgical operation.
